# Testing the robustness of Citizen Science projects: Evaluating the results of pilot project COMBER

**DOI:** 10.3897/BDJ.4.e10859

**Published:** 2016-12-21

**Authors:** Giorgos Chatzigeorgiou, Sarah Faulwetter, Thanos Dailianis, Vincent Stuart Smith, Panagiota Koulouri, Costas Dounas, Christos Arvanitidis

**Affiliations:** 1 Institute of Marine Biology Biotechnology and Aquaculture, Hellenic Center for Marine Recearch (HCMR), Heraklion, Crete, Greece; 2 University of Patras, Department of Biology, Laboratory of Zoology, Rio, Patras, Greece; 3 The Natural History Museum, London, United Kingdom

**Keywords:** Coastal biodiversity, Bio-watch, representativeness, robustness

## Abstract

**Background:**

Citizen Science (CS) as a term implies a great deal of approaches and scopes involving many different fields of science. The number of the relevant projects globally has been increased significantly in the recent years. Large scale ecological questions can be answered only through extended observation networks and CS projects can support this effort. Although the need of such projects is apparent, an important part of scientific community cast doubt on the reliability of CS data sets.

**New information:**

The pilot CS project COMBER has been created in order to provide evidence to answer the aforementioned question in the coastal marine biodiversity monitoring. The results of the current analysis show that a carefully designed CS project with clear hypotheses, wide participation and data sets validation, can be a valuable tool for the large scale and long term changes in marine biodiversity pattern change and therefore for relevant management and conservation issues.

## Introduction

A sharply increasing trend in data being gathered by citizen scientists (CSs) without any scientific background has been recorded in the last couple of decades in many different scientific issues. This trend has tremendously facilitated by the platforms developed on the internet, the applications for mobile phones and finally by the popularity and the scope of the SCs activity (Curtis 2014). Three factors seem to be responsible for the "explosion" of this activity: (a) the existence of easily available platforms for disseminating information on these projects and the techniques for gathering data; (b) the increasingly growing confidence among the scientists that the public represents a free source of labour, skills, and even funding; (c) in addition, CS is likely to benefit by research funding agencies, which now suggest, if not impose, upon every grant-holder to implement such a project-related outreach activity (Johnson et al. 2014). The primary hypothesis behind these projects is that CSs can collect a vast amount of data and information which the scientists wouldn't be able to collect otherwise because of time and resources limitations (Dickinson et al. 2010). The benefit for the citizens, on the other hand, can be their motivation to contritube to the real world of science, public information and conservation (Silvertown 2009). It is widely accepted that in the absence or the high cost of automatic sensors, CS projects can help to servey biodiversity in wide spatio-temporal coverage. Long term range and large geographic extent are required to document biodiversity pattern changes and to address relevant questions at the scale of the species spatio-temporal coverage (e.g. migrations) as well as to implement new policies on a national or regional level and mitigate the impacts of environmental processes like climate change (Tulloch et al. 2013). Until recently, there are several cases in which decision makers used the CS data and information to change policy at national scale and to take action for environmental conservation issues (Couvet and Prevot 2015).

Hundreds of thousands of CSs daily participate to projects related to climate change, invasive species, conservation biology, ecological restoration, water quality monitoring, population ecology and biodiversity monitoring (Silvertown 2009). Traditionally CSs projects deal with a wide variety of species like birds, butterflies, mammals etc (bird-watching: ebird.org, butterfly-watching: monarchwatch.org, whales watching: whalewatching.org, etc.). However, this tendency is still rare in the marine habitats (Cigliano et al. 2015).

The CS projects can be divided into two broad categories: (a) those that they have a scientific approach for the data collection at their hardcore, that is at all those steps from a purely scientific question to the analysis of the data and interpretation of the results and, (b) those that primarily work to address education, outreach and awareness purposes (Loss et al. 2015).

Despite the willingness of the individual citizen scientists to collect many data, a crucial point for the success of any CS project is the quality of the datasets they create. One of the main challenges of CS projects from scientific and policy perspective is the quality of the data in terms of accuracy and precision, spatial and temporal resolution, robustness, documetation, and access (Hyder et al. 2015). Many studies have been testing whether the quality of CS created data sets meet the standards required to address the scientific questions in terms of quality and reliability or they are unreliable for the purposes of the research project (Tulloch et al. 2013). Results of those studies reveal that with the proper treatment, data sets may become a useful and reliable tool for both the scientific community and the decision makers (Paul et al. 2014).

More than 200 CS projects are nowadays active (Cigliano et al. 2015) and, although most of them have been established over the last decade, some of these projects are running for more than a century. From the totally active CS projects only the 14% of them refer to marine habitats, most probably because marine CS projects encounter challenges not faced in the terrestrial ones (Cigliano et al. 2015). The major problem is the habitat accessibility where citizens, at the best, spend only a part of their lives. This fact significantly reduces the number of volunteers and visits/records on the marine species and habitats, and this has as a result the limited number of available data sets. In many cases, access to marine habitats often require expensive equipment like boat, diving gear, transportation etc, which bring additional limitations (Roy et al. 2012, Theobald et al. 2015). Moreover, many cultures and societies have not yet adapted to swim or incorporate marine activities into daily life and thus training is needed in order to shift the public interest onto the marine environment (Cigliano et al. 2015). Finally, species identification and habitat delimitation is not always a straight forward process as visibility and colour pattern of species vary and habitat boundaries or their transitions may be missing in several cases. Furthermore, visibility and colour patterns become less bright as the depth increases. The above factors, among others, are the main reason why marine and coastal CS projects are under-represented (Cigliano et al. 2015). However, the development of low-cost housing gear for digital cameras has increased the number of pictures that one can take which is only limited by the time spent underwater and the time to sort and index them. With the development of cloud solutions, the storage is not an issue any longer. Furthermore, there are several marine CSs projects in which scuba divers are used as “oceanographic samplers” with the view to collect environmental data such as sea temperature (Wright et al. 2016).

A pilot CS project named COMBER (Citizens’ Network for the Observation of Marine BiodivERsity, http://www.comber.hcmr.gr) has been established in the region of the eastern Mediterranean Sea in the context of the ViBRANT (Virtual Biodiversity Research and Access Network for Taxonomy: http://vbrant.eu) project. It has been designed for divers and snorkelers who are interested in participating to coastal marine biodiversity projects. The project aimed at engaging CSs in a marine coastal biodiversity observation network. The main scientific objective of the project has been to test the willingness of SCUBA divers (fun divers or diving club members) to join the project and the quality of the collected data and relevant information.

Therefore, the purpose of this study is to test the robustness and the representativeness of CSs data sets collected in the course of the COMBER project in order to address the marine coastal biodiversity monitoring task based on a single taxon: The most common coastal fish species of the eastern Mediterranean, which is used as a proxy in order to test two hypotheses: (a) The H_0_ scientific hypothesis on the robustness of the data sets collected during the project, is that there are no differences between the levels of experience of the identifier (CS diver) in the emerging multivariate patterns; in other words, emerging multivariate patterns are independent from the CS data collector and his/her experience; (b) On the issue of the representativeness, the H_0_ scientific hypothesis, in order for the data collected to be valuable for biodiversity assessment studies, is that the fish species lists of the samples collected by each diver, along with their higher classification (as an approximation of the phylogenetic/taxonomic diversity of a sample), should be random assembly of the broader fish inventories from regional pools. In this way randomness is used a a means to infer representativeness of the collected datasets.

## Materials and Methods

### Project Description

The pilot COMBER project has been operating in the Aegean Sea, primarily focusing on the Cretan shallow (less than 50m deep) marine habitats, with the potential to expand the concept into the whole Mediterranean basin or any other region (Arvanitidis et al. 2011). The fish species inhabiting these shallow habitats have been chosen as the target taxon for the project implementation since it is one of the most common taxa in Mediterranean shallow habitats and one of the most attractive to the divers and snorkelers. For the underwater species identification, the waterproof "bio-watch" fish identification card (http://www.bio-watch.com) is used. This card presents fourty of the most common fish species in Mediterranean shallow habitats on a pictorial key based on morphological features, colour pattern and habitat (Dounas and Koulouri 2011). The COMBER project focuses mainly on two target groups of volunteers: a) people skilled to dive with mask and snorkel and b) certified scuba divers. The dissemination of the project is achieved by: a) the web site of the project; b) information desks; c) posters and leaflets which are distributed in the participated diving clubs and information tourist offices. In some cases participants have been approached directly at the diving clubs just before their dive. Each participant has been equipped with a fish card which is used both to identify the species and to keep notes for the observed species.

### Data collection

The participants had the option to keep notes for their observations in two ways: presence/absence and relative abundance of four different orders (blank: absent, one bar: 1-3 individuals, two bars: 4-10 individuals and three bars: more than 10 individuals). A short seminar has been provided before each dive excursion, with the view to introduce the SCUBA divers into the concept of the specific CS project. A seminar, of a fifteen minutes presentation, explaining the way of identifying the fish species on the card and how to record them has been delivered before the dive. At the beginning of the dive and for the first ten minutes, participants assisted by the scientists are identifying the different species they can observe and which are depicted on the fish card without recording them. After this ten minutes period, the divers identify and record by themselves. A small de-briefing is following after each dive and all the participants are kindly requested to fill in a questionnaire regarding their past and present experience. Finally, the participants are guided to navigate in the website, create an account, fill in their diving profile and enter their species observations into the database (see for the detailed description of the process in Arvanitidis et al. (2011)). This pilot project has been running over three consecutive years in cooperation with two diving clubs and one sailing school.

### Statistical analysis

In order to convert the four orders of abundance into numerical values, a new rank with four orders of magnitude was created: absent: 0, one bar: 10 individuals of a certain species, two bars: 100 and three bars: 1000. Additionally, in order to down-weight the differences in divers effort, the abundance values in the recordings of each diver (unique ID) were averaged and then the triangular similarity matrices were produced by applying the Bray-Curtis coefficient using the divers data (rows = species; columns = divers; cells = data). To test the first hypothesis, the multivariate patterns of fish card species distribution were derived by using the algorithm of the non-metric multidimensional scaling (nMDS) on the corresponding similarity matrices as proposed by Clarke and Warwick (1994). Subsequently, an analysis of similarities (ANOSIM) was applied to determine the level of (dis)similarity between the *a priori* defined data collecting groups, that is those differing in (i) identification experience, (ii) diving experience and (iii) implementation year. In the first category, the knowledge background was divided into three major categories: (a) Amateur, for those divers without any previous experience in species identification, (b) Skilled, for those with some previous experience in species identification and (c)Professional, for those with a great experience in species identification. In the second group (diving experience) and independently of their species identification capacity, the participants were divided according to their diving experience into: (a) Novice (up to 20 dives), (b) Intermediate (between 21-50 dives) and (c) Experienced (more than 51 dives). In the third category (implementation year) the dataset was divided into the years of project implementation (2011-2013).

The ANOSIM test calculates a sample statistic R with values ranging between -1 and 1 (usually 0-1), where R = 1 represents an undeniable difference between the groups under comparison. The application of the routine in the PRIMER package provides a simulated distribution of possible R values on a frequency histogram and superimposes the observed value on that histogram. Observed R values outside the expected distribution are taken as statistical evidence to reject the null hypothesis (no differences between groups).

### Randomization Test

To test the second hypothesis, that is whether the fish species lists recorded by the divers, based on the use of the fish-card (that is the most common eastern Mediterranean shallow fishes), are randomly assembled from the fish species pool of the broader area, a hierarchical approach was applied. Six different scales of observation are defined after an extended literature research: (a) Mediterranean fish inventory (b) Eastern Mediterranean fish inventory, (c) Aegean fish inventory, (d) down to 50m depth Mediterranean fish inventory, (e) down to 50m Eastern Mediterranean and (f) down to 50m Aegean. The last three of the aforementioned scales were specifically chosen because of the use of the "bio-watch" fish card created for divers and snorkelers and thus all the included species live in shallow waters and therefore are observable. For the construction of observational species list the data base from FishBase was used (www.fishbase.org). The test was run for the two different categories of data set based on: a) diving experience and b) identification experience. At each scale of comparison it was tested whether the biodiversity observation subsets, which means the species lists recorded by the divers and their higher phylogenetic interrelations, represent a random sample of the higher observational scales. The above test was performed by calculating the taxonomic distinctness indices (average taxonomic distinctness, Δ^+^ and, variation in taxonomic distinctness, Λ^+^). These indices take into account not only species ID but also their phylogenetic / taxonomic interrelations (e.g. Warwick and Clarke 2001). By this method, the 95% funnel-shaped confidence limits of the expected distribution of values were calculated from the respective higher observational scale through permutations, and the observed values from the samples of the fish card, that is the recorded species lists by the divers, were then superimposed on these funnel-shaped confidence limits. Hence, if the samples were located within the funnel limits they were considered as random samples of the higher observational scale. In contrast, if the samples were located outside the funnel limits, this was taken as statistical evidence that the observed species in the lists are more closely related to each other than expected if they were assembled at random (further information about the randomization test can be found in Somerfield et al. 2009).

The theoretical background for this approach is based on Warwick and Clarke (1995) concept which claims that in stressful conditions species assemblages tend to be more closely related to each other than expected because they're obliged to respond to the same disturbance factors by developing the same strategies and thus by sharing in common similar characters. Thus the results of randomization test, at least to some extent, will reveal if the collected datasets are representative of the broader area and if so it means that there is some scientific value in them.

## Results

In total 141 divers and snorkelers (unique ID) have participated in the COMBER pilot project. The participants have submitted 365 data sets (5,600 observations) within the three years of the pilot project implementation. More than half of the participants (61%) contribute to the database at least twice, with the highest record 28 entries from the same participant. The most common species found to be the *Coris julis* (256 / 365) while the less abundant was *Raja clavata* (1/365).

### Do groups of observations differ?

The MDS plots (Fig. 1 a,b) illustrate the similarity between all data sets, that is all species lists recorded by the participants (i) based on identification experience, (ii) on diving experience and (iii) on project implementation years (not shown). It is obvious on the MDS plots that the data sets produced from experienced users in both cases appeared to be less scattered and concentrated at the centre of the plot. In addition, ANOSIM tests have shown that in all cases no significant differences were recorded (Table 1).

### Is the dataset representative and if yes to what extent?

A summary of the results from the tests for both taxonomic distinctness indices (Δ^+^, Λ^+^) at all scales of observation are shown in (Fig. 2 a, b). In the case of Δ^+^ the majority of the data sets fall outside and below the 95% confidence limits while in case of Λ^+^ almost the total of the data sets fall inside the 95% funnel limits. This pattern seems to be steady no matter the observation scale or the experience level of the participant divers.

## Discussion

Citizen science projects are booming undoubtedly, however the crucial question about the reliability of the datasets has not yet been fully clarified. The scientific effort of COMBER project attempts to shed some light onto whether the collected data sets have a scientific value and if yes to what extend (e.g. Bird et al. 2014, Burgess et al. In Press).

The performance of the MDS technique produces a broadly scattered and without a clear distinction pattern between the datasets collected from the two broad categories of analysis (diving and identification experience) and between the years of the project initial implementation. The analysis of similarities shows that there are no statistical differences between the produced data sets in reference with the collection years, diving experience and identification skills. At this point must be underline that the stress values on both analysis (diving and identification experience) were greater than the crucial value 0.2. Based on Kruskal (1964) values greater than 0.2 means but fit. Nevertheless stress value by its self gives a vague indication of the goodness of fit. In addition stress increases both with the number of samples and with the number of variables and in case of Comber data set the number of samples were 350. Consequently, in case of COMBER the data sets seem to be independent from the skills of the data providers. The degree of independence of produced data sets is an important part of CS projects success and in the COMBER case this independence can be found at all scales of analyses.

With the analysis of representativeness we test whether the species lists from fish card in the collected data sets are representative of the Mediterranean region. The results from taxonomic distinctness indices have shown an opposite pattern: (a) The Δ^+^ funnels show that at all levels of observation and for all different categories of divers the majority of the datasets fall outside the expected distribution; (b) This pattern is altered in case of Λ^+^ index funnels, where the majority of the participant’s datasets fall inside the funnels limits. Taking into account the theoretical background and the mathematical formula of the two indices it’s easy to explain the converse patterns obtained from both of them. There is an undeniable bias in the fish card species list and their higher classification since at least two fish families (Sparidae and Labridae) are over-represented. These two families are, indeed, very common in the shallow coastal Mediterranean waters (Ruiz-Frau et al. 2011) and they can be identified relatively easy. The over-representation of those two fish families though causes a distortion in the phylogenetic dendrogram and reduces the values of Δ^+^ index. Contrarily, the same feature of the fish card changes dramatically the distribution of the species to the higher taxa and this is reflected as an uneven distribution of the branches of the total phylogenetic tree. This unevenness increases, on the other hand, the values of the Λ^+^ index and that is the reason why almost all datasets fall inside the funnel limits. Despite this over–representation of the two families, the datasets generated by the fish card species list seem to be representative of the Mediterranean Sea for all scales of observation, as well. This is the first time that datasets from a marine CS project are tested under this view.

Questions on large scale long term biodiversity patterns and their changes can be answered through CS projects. The collection of such data by exclusively scientists requires a vast amount of budget and effort. In addition, the large number of publications (US Breeding Bird Survey and Christmas Bird Count have resulted in over 500 and 300 publications, respectively; (Evans 2013)) provides strong evidence on the scientific value of information derived from CS projects. Although these data have received strong criticism by many scientists about their value and quality, the emerging problems can still be resolved with good experimental design, adequate training of amateur scientists, ground truthing, model parameterization and metadata analyses tests (Bird et al. 2014, Burgess et al. In Press). In addition, the gap between the social and environmental scientists can be limited by figuring out how citizen science concept affects human understanding behavior (Cooper et al. 2007).

Citizen’s participation in marine CS projects is limited comparing with the terrestrial one. The main reason for this “unbalanced representation” is the accessibility and the associated costs, which are more direct and lower, repectively, in the case of terrestrial activities. Volunteers who dropping out or becoming disinterested could possibly be convinced to come back with some degree of positive reinforcement (i.e., informing them how they are impacting conservation) (Whitelaw et al. 2003, Legg and Nagy 2005), by matching monitoring protocols to their specific interests and skills (Whitelaw et al. 2003) or by receiving a feedback for their contribution as a reward for their participation. However, the diﬃculties and the expertise needed for species identification and monitoring has led many scientists to believe that CS data collection programs tend to have higher value for higher taxonomic levels because of species identification problems (e.g. Kremen et al. 2011). Thus, collected data by participants must be validated in some way. For this reason, modern analytical approaches have been developed which can account for many types of error and bias, typical of the CS datasets (Bird et al. 2014). The results of this study show that the hypotheses to be tested by data collected by CSs have to be clearly stated from the beginning of the project in order to minimize all the speculations or misleading points.

At this point, it must be underlined that the citizen scientists have to be categorized in different groups, based on their contribution in the projects: a) basic level, where participants contribute only in data gathering, b) advanced level, where participants, may make suggestions and try to improve the purpose of the project and, c) professional level, where participants are involved in the setup of the project (Bonney et al. 2009). The results of those volunteer categories have to be treated in a differently weighted way since the confidence level of collecting data is different.

To conclude, CS projects become an increasing need for biodiversity monitoring by collecting large scale, long term data. Until now, the results of existing projects have shown that the collected data have to some extent an important scientific value if they are analyzed in the proper way. Most of the already running projects show that volunteers are able to detect important changes in communities through their data and so they have a valuable role to play in assessing change on biodiversity and ecosystems (Forrester et al. 2015). Accordingly, COMBER provides important results on the concept and the implementation of this category of projects. However, only with the expansion of the project in other public groups like recreational divers and the creation of a much larger data series from all over the Mediterranean, safer conclusions may be drawn.

## Figures and Tables

**Figure 1a.**
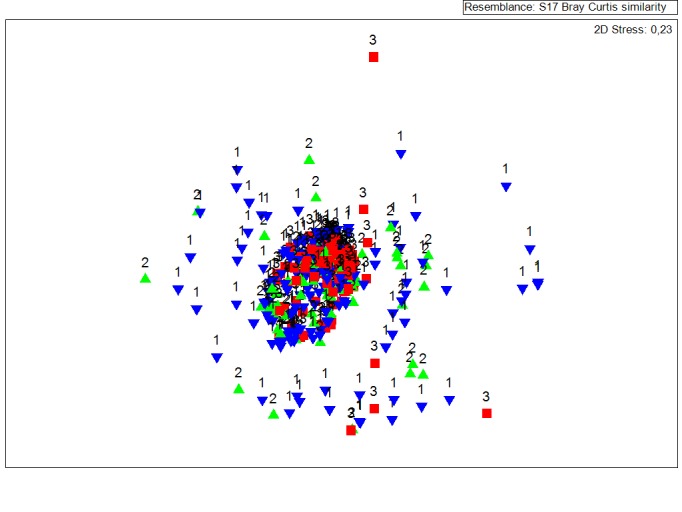
Identification Experience

**Figure 1b.**
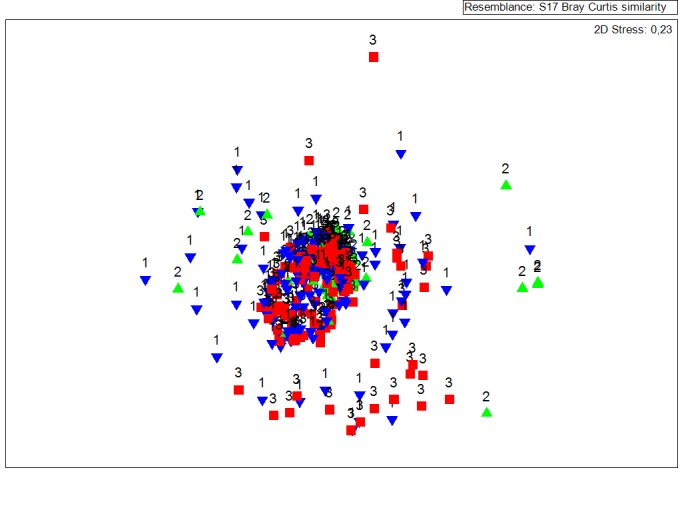
Diving Experience

**Figure 2a.**
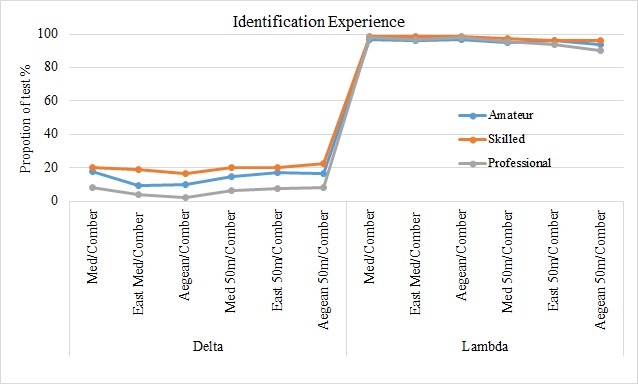
Identification Experience

**Figure 2b.**
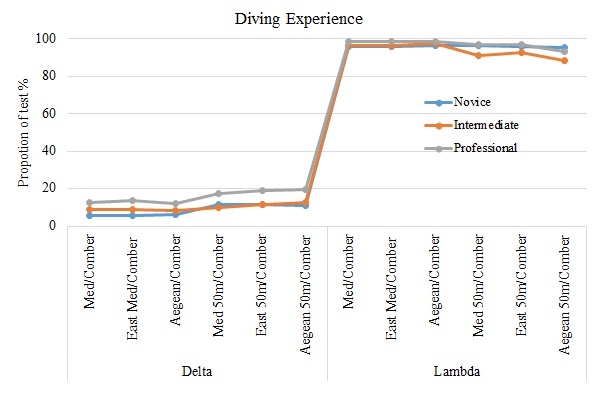
Diving Experience

**Table 1.** Results of the one-way ANOSIM testing for differences among Factors: Diving Experience (Goup1: Amateur, Group2: Skilled, Group3: Professional), Identification Experience (Goup1: Novice, Group2: Intermediate, Group3: Experienced) and Years of Implementation (Goup1: 2011, Group2: 2012, Group3: 2013)

	Diving Exp		Identification Exp		Years Impement	
Factors	R value	*P*	R value	*P*	R value	*P*
Total	0.015	0.172	0.057	0.341	0.115	0.396
Group1 , Group2	0.014	0.173	-0.036	0.222	0.034	0.329
Group2 , Group3	0.016	20.8	0.214	0.231	0.235	0.365
Group3 , Group1	0.006	0.308	0.55	0.421	0.12	0.317
